# Reaching consensus: a scoping review on erectile disorder guidelines

**DOI:** 10.25122/jml-2022-0106

**Published:** 2022-09

**Authors:** Minoo Safaei, Raziyeh Maasoumi, Seyed Amirhosein Mahdavi, Laleh Ghadirian, Keshvar Samadaee Gelehkolaee

**Affiliations:** 1 Department of Reproductive Health, School of Nursing and Midwifery, Tehran University of Medical Sciences, Tehran, Iran; 2 Nursing and Midwifery Care Research Center, School of Nursing and Midwifery, Tehran University of Medical Sciences, Tehran, Iran; 3 Legal Medicine Research Center, Legal Medicine Organization, Tehran, Iran; 4 Knowledge Utilization Research Center, Center for Academic and Health Policy, Tehran University of Medical Sciences, Tehran, Iran; 5 Men's Health and Reproductive Health Research Center, Shahid Beheshti University of Medical Science, Tehran, Iran

**Keywords:** guideline, guidance, erectile dysfunction, erectile disorder, sexual disorder, sexual dysfunction, male sexual dysfunction, male sexual disorder

## Abstract

Erectile disorder (ED) is the continuous or repeated inability to achieve an erection or maintain its firmness for an adequate amount of time during sexual intercourse. Given the importance of utilizing quality clinical practice guidelines (CPGs) for the diagnosis of ED, the research team conducted a scoping review of erectile disorder CPGs to address the questions based on the clinical guideline of the best quality. This scoping review was conducted in five steps: 1 – identification of the research question, 2 – identification of relevant studies, 3 – selection of studies, 4 – data extraction, 5 – summarizing and reporting the results. The initial search yielded 1,888 articles, CPGs and books but after primary and secondary screening by two appraisers, 9 CPGs were extracted. After that, the two appraisers examined the quality of these guidelines using the AGREE II tool. Eventually, 5 CPGs extracted. After reviewing 5 guidelines, 5 questions were answered. Overall, the CPGs had desirable overlap in response to the marked questions; nevertheless, there were some differences in details too. This review shows that the first and foremost principle in examining the affected individuals is taking a complete detailed history, followed by a physical examination and use of relevant questionnaires to complete the information necessary to diagnose the problem. The next step is to perform routine lab tests; hormonal profiles may also be checked, and if necessary, special tests should be performed based on an individual's conditions.

## INTRODUCTION

In general, male sexual dysfunction is regarded as any change in at least one phase of the sexual response cycle (desire, arousal, orgasm, resolution) [1]. Based on the DSM-5 classification, male sexual dysfunctions include erectile disorder, hypoactive sexual desire disorder, premature (early) ejaculation, delayed ejaculation, substance/medication – induced sexual dysfunction, and other specified or unspecified sexual dysfunctions [2]. Penile erection results from the appropriate function and coordination of nervous, vascular and hormonal systems on relevant male genital tissues [3, 4]. Erectile disorder is the continuous or repeated inability to achieve an erection or maintain its firmness for an adequate amount of time during sexual intercourse [3, 5–7].

Erectile disorder (ED) may have many causes: vascular, nervous, local factors related to the penis, hormonal, medication or psychologically – induced [6–8]. It may also be a sign of underlying health problems. It may be the first sign of cardiovascular disease or peripheral vascular disease [2, 5, 9]. Some causes of ED have psychological roots, such as: stress, depression, functional anxiety, pornography addiction and relationship – related concerns [3, 4, 10]. Epidemiologic data indicate a high prevalence of ED across the world [3, 11]. Twenty percent of the male population are affected by ED. The prevalence of ED in men aged over 40 is 53%, the percentage of which increases with increasing age [4, 6, 12]. These conditions significantly affect the patient and his sexual partner's sexual health [3, 5] and becomes a strong source of anxiety [5]. The diagnosis and management of ED is of concern for both physicians and patients, as in addition to its high prevalence, it deeply affects couples' sexual quality of life as well [6].

Although sexual problems are very common, these cases are often overlooked and are left undiagnosed. Moreover, it has been said that physicians are not properly familiar with the approach to detect and assess sexual problems. It is often suggested that psychiatrists and related specialists should have extensive knowledge on sexual issues and adopt an appropriate approach toward them [13]. General physicians, urologists, internal specialists, psychiatrists and other healthcare personnel and experts should be encouraged to talk to men about sexual issues to detect those affected with ED and other sexual disorders. Otherwise, patients might not voluntarily speak about their sexual problems and concerns [14]. The basic concept of sexual dysfunction management is to adopt a patient–centered framework for assessment and treatment [13]. Given the variety of clinical measures, clinical guideline developers and urology-, sexology-, and other relevant scientific associations have designed clinical practice guidelines using different methods [12]. The goal of erectile disorder management clinical practice guidelines is to provide clinical physicians with a guide to give correct consultations to men with ED and help them maintain and promote their quality of life. These guidelines are focused on the diagnostic hierarchy of ED, how to perform a valid diagnostic procedure, and to achieve treatment with the goals of sexual function recovery, promoting the patient and his partner's quality of life, as well as minimizing the side effects of treatment [13, 15]. Clinical practice guidelines (CPGs) are not only a reference for health experts, but also a source of information for patients and policymakers in many countries and have been developed to provide valid recommendations to decision-makers [12] and strengthen the patient-physician relationship. CPGs state the methods of clinical handling of the patient in a stepwise fashion bearing in mind the priorities, effectiveness and cost-effectiveness of various treatments [9]. The benefit of CPGs depends on their quality. The utilization of appropriate methods and accurate strategies in the clinical guideline development process is very important for the successful implementation of the recommendations. The quality of these guidelines can be very variable and some of them are lower than basic standards [16]. Given the importance of utilizing quality CPGs for the diagnosis of ED, the research team conducted a scoping review of erectile disorder CPGs to address the questions based on the clinical guideline of the best quality.

## MATERIAL AND METHODS

Recently, the utilization of scoping review is more often seen in the field of evidence synthesis. Since these types of studies follow a regular structural process, they are like systematic reviews. Nowadays, scoping reviews are recognized as a valid method when systematic reviews are unable to meet the users' demands or goals [17]. Scoping reviews are for situations where in it is unclear what other specific questions can be raised and a more accurate systematic review for responding to these questions needs to be done [18]. This study was conducted in the form of a scoping review to respond to basic questions in the field of ED. Scoping review is a type of knowledge analysis that rapidly examines the key concepts of a specific research topic and finds the main resources and types of evidence [19]. A scoping review study includes five steps: 1 – identifying the research question; 2 – identifying relevant studies; 3 – study selection; 4 – data extraction and 5 – summarization and data reporting [20].

### First step: Identification of the research question

The research identification question is based on PICO. PICO consists of four components: intervention, population, the compared method, and the outcome [21]. In this study we used three components of PICO, including: population (men, man, human), intervention (guideline, guidance, protocol, instruction, management, clinical practice), and outcome (recognition, diagnosis, assessment). The importance of designing the research question in this framework is that this framework includes all the key components of clinical decision making and its structure is similar to the structure of clinical research evidence [22].

### Second step: Identification of relevant studies

In this study, we determined the inclusion and exclusion criteria and the databases for searching studies. Moreover, we also used databases dedicated to male sexology and sexologists' opinions.

#### Inclusion and exclusion criteria

The inclusion criteria were, being published during the specified time-period (2015–2020), relevancy to the research topic, being in English or Persian, and the most recent version of the clinical guideline. The exclusion criterion was the unavailability of the clinical guideline's complete version.

#### Databases

Search was conducted using the relevant keywords and their synonyms in search engines dedicated to guidelines, select databases, and databases dedicated to male sexology (Table 1). MeSH was used for the keywords.

**Table 1:** The list of search engines used for clinical practice guidelines on erectile disorder.

Search engines dedicated to guidelines	Databases	Databases dedicated to male sexology
Guidelines International Network (GIN); National Institute for Health and Clinical Excellence (NICE); Trip Database; NICH; Cochrane reviews and Cochrane protocol; UpToDate.	Web of Science; Scopus; PubMed; Google scholar; Embase.	American Urological Association; European Association of Urology; Canadian Urological Association; Colombian Society of Urology; Mexican Society of Urology; Argentinian Society of Urology; Brazilian Society of Urology; Spanish Association of Urology; International Society for Sexual Medicine; World Association for Sexual Health; European Society of Sexual Medicine; Healthy Male (Andrology Australia).

#### Keywords

Guideline, guidance, protocol, instruction, management, clinical practice, erectile dysfunction, erectile disorder, sexual disorder, sexual dysfunction, male sexual dysfunction, male sexual disorder.

### Third step: Study selection

The CPGs were separately assessed by two appraisers from the research team in two steps. In the first step, the titles and abstracts were screened, and in the second step, the full texts were screened for the inclusion and exclusion criteria. During the first phase, the CPGs that had relevant titles were included in the research, and in the second phase the full texts of the CPGs were separately examined by two appraisers for the inclusion and exclusion criteria.

The AGREE II (The Appraisal of Guidelines for Research & Evaluation) critical appraisal tool can be used to examine the quality of the CPGs. AGREE is meant for solving the problem of variety in the quality of developed CPGs. The goal of this tool is to provide a framework for assessing the quality of CPGs. The use of this tool in the quality assessment of CPGs is preferred globally, given its comprehensive nature and focus on various aspects of assessment. AGREE consists of 23 key criteria that are classified into six sections: scope and purpose, stakeholder involvement, rigor of development, clarity of presentation, applicability, and editorial independence. Each section deals with one aspect of guideline quality [23]. The validity and reliability of this tool has been approved in earlier studies [24].

Here, the CPGs' quality was evaluated by two appraisers separately using the AGREE II instrument. Each section's score is obtained by summing up the scores given to its criteria and standardizing the overall score, given the maximum score attainable in that section. The tool's six sections' scores are independent of each other and should not be added to give a total score to the guideline.

Upon evaluation, each of AGREE II domains' scores were calculated as follows: (Obtained score − minimum possible score)/(maximum possible score − minimum possible score).

### Fourth step: Data extraction

Based on the study objectives, the data extracted from the CPGs were classified in response to five questions: 1 – What is the first step in assessing erectile disorder?; 2 – What are the principles of examining patients with erectile disorder?; 3 – Which questionnaires are better to use for assessing erectile disorder?; 4 – What lab tests are appropriate for men with erectile disorder?; 5 – Which special tests are better to use for men with erectile disorder?

### Fifth step: Summarization and data reporting

In this scoping review nine guidelines which possessed the inclusion criteria were examined. We used five of these that had scored higher in the domains based on the AGREE II tool (Table 2). What follows is the detailed description of the questions' responses.

**Table 2:** Standardized scores obtained by guidelines appraised by AGREE II.

Guideline name	Institute	Year	Domain 1 score	Domain 2 score	Domain 3 score	Domain 4 score	Domain 5 score	Domain 6 score
Evaluation of male sexual dysfunction	UpToDate 2020	2020	61.1%	75%	31%	100%	11%	50%
Erectile dysfunction: AUA Guideline	American Urological Association 2018	2018	89%	75%	93%	87%	33%	100%
British Society for Sexual Medicine guidelines on management of erectile dysfunction in men-2017	International British Society for Sexual Medicine 2017	2017	89%	67%	62%	71%	72%	50%
Practice guidelines for erectile dysfunction	Canadian Urological Association 2015	2015	94%	58%	50%	75%	44%	92%
EAU Guidelines on erectile dysfunction, premature ejaculation, penile curvature and priapism	European Association of Urology 2019	2019	100%	79%	79%	83%	55%	42%
Erectile dysfunction diagnosis and management	Healthy Male Andrology Australia 2018	2018	50%	25%	0%	58%	0%	0%
Clinical practice guidelines for management of sexual dysfunction	Official publication of the Indian Psychiatric Society	2017	89%	54%	21%	58%	33%	8%
Clinical Practice Guide in Erectile Dysfunction	Malaysia Urological Association and MEDACT	2017	78%	58%	26%	79%	44%	17%
Investigation and management of erectile dysfunction and male hypogonadism	Toward Optimized Practice	2016	89%	46%	28%	92%	28%	0%

## RESULTS

**Figure 1:**
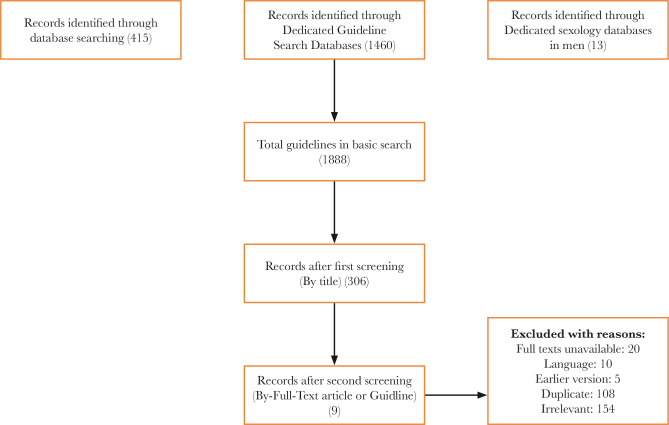
Result of the scope review search.

The initial search of the databases, search engines dedicated to guidelines, and databases dedicated to male sexology yielded 1888 articles, CPGs and books. Primary screening was done by two appraisers based on relevant titles and clinical guideline summaries, which led to the selection of 306 CPGs for further evaluation. In the next step, the guidelines were examined for possessing the inclusion criteria and their full texts were studied, which led to the final inclusion of 9 CPGs (Figure 1). Thereafter, the two appraisers examined the quality of these guidelines using the AGREE II tool. Eventually, 5 CPGs published in UpToDate (2020), American Urological Association (2018), International British Society for Sexual Medicine (2017), Canadian Urological Association (2015), and the European Association of Urology (2019) that had higher quality were selected to respond to the questions (Table 2).

### Question 1: What is the first step in assessing erectile disorder?

Taking a diagnostic history is key in assessing sexual dysfunction and erectile disorder [14]. An accurate sexual history is the first step in the assessment of men with ED [8]. It is to obtain an accurate medical, sexual, psychological and social history of patients and if possible, their spouses as well [9, 15]. A detailed history of the problem, including the duration of symptoms and the main issues associated with them should be obtained [25].

### Question 2: What are the principles of examining patients with erectile disorder?

The principles of examining patients with erectile disorder include taking a complete medical, sexual, psychological and social history. If there is reason to believe that physical reasons are the cause then focused physical examination and lab tests (routine and special) should be done [8, 9, 15, 25]. Furthermore, the Canadian Urological Association (2015) also recommends a fourth principle, which is to consult with specialists appropriate to the patient's problem [14].

### Question 3: Which questionnaires are better to use for assessing erectile disorder?

Mostly, the International Index of Erectile Function (IIEF) is used to examine ED [8, 9, 25]. Other scales have also been named, such as the Sexual Health Inventory for Men (SHIM) [9, 25]. SHIM assesses different domains of sexual function (sexual arousal, erectile function, orgasmic function, intercourse and overall satisfaction), and helps examine the potential impact of a special therapeutic method. Using the International Prostate Symptom Score (IPSS) and the Aging Males' Symptoms (AMS) scale score may also prove helpful in assessing sexual function domains and the effect of treatments and interventions, but they are no substitute for complete history taking and physical examination [25].

### Question 4: Which lab tests are appropriate for men with erectile disorder?

Since ED is closely associated with underlying diseases, be it implicitly or explicitly, all those complaining of this disorder should be examined for underlying diseases. Appropriate lab tests for men with ED usually include: fasting blood glucose or HbA1C for the testing of diabetes or glucose control levels, complete blood count, complete metabolic profile for the assessment of hepatic and renal functions, thyroid stimulating hormone (TSH) to rule out thyroid disease, lipid profile to assess cardiac risk factors, and total serum testosterone to assess sexual glands [8, 9, 14].

The levels of live or free testosterone may be needed to confirm the total testosterone level measurement. Fasting serum testosterone should be measured between 8 to 11 am. Free serum testosterone is a reliable measurement of androgen status and is highly correlated with clinical symptoms, however, often, total serum testosterone levels are available. If the serum testosterone level is borderline or low, the morning sample should be measured again along with serum LH and prolactin levels (total testosterone <8 nanomoles/liter) [9, 25]. Additional lab tests may be considered in patients (*e.g*., prostate specific antigen (PSA), prolactin, and luteinizing hormone (LH) [9, 25].

### Question 5: Which specific tests are better to use for men with erectile disorder?

Based on select CPGs, the tests specific to men with ED include: nocturnal penile tumescence (NPT) test, duplex doppler imaging, intracavernous injection test, arteriography, dynamic infusion cavernosometry or cavernosography (DICC) [8, 9, 14, 15]. We will explain the indications of using each of these below.

#### Nocturnal penile tumescence (NPT) test

The NPT test has been mentioned as the first test [8, 9, 14, 25]. This test is applicable for differentiating between psychological and physical causes of ED, given that men with psychogenic ED can have nocturnal erections too [8, 15]. It is also used when other tests have failed or before the surgery [4]. Some CPGs consider the greatest application of this test to be pharmacological research studies have used it to assess the effect of treatment [14].

#### Penile duplex doppler sonography

The penile duplex doppler sonography helps the physician better understand the cause of ED [8]. Other indications for performing the penile ultrasonography include: penile trauma, priapism, prion disease or unresponsiveness to phosphodiesterase inhibitors and other drugs [8]. If the duplex sonography test result is normal, then no further vascular evaluation is necessary [9, 14]. Currently, it is the gold standard for assessing penile vasculature and is considered the least invasive, providing strong information on intracavernous arterial flow and penile venous obstruction capacity [15]. The resultant data from this test may be useful for the following:

- Differentiating between the physical and psychogenic causes of ED;
- Assessing arterial function in men, which might be performed by a cardiologist;
- Identification of men affected with severe venous obstruction, the disordered function of which leads to ED, and which is unlikely to respond to medical treatment;
- Identification of young men who may be candidates for penile revascularization [15].

Evidence also suggests that findings of penile duplex doppler sonography can predict endothelial function disorder [25].

#### Intracavernous injection test

The intracavernous injection test does not provide a diagnostic result and, if approved clinically, a duplex doppler sonography must also be done on the penis [9]. Its application as a diagnostic test in ED is limited, as positive results can be seen in both normal individuals and patients with mild vascular disorders. The main application of this test is in assessing the change in penile shape, to help plan the surgical management [25]. This test is less conducted since the emergence of effective oral medications in Canada [14].

#### Arteriography, dynamic infusion cavernosometry or cavernosography (DICC)

Arteriography and DICC should only be performed in patients who are undergoing vascular reconstruction surgery [15] and/or if the doppler assessment shows arterial insufficiency [2, 5]. Cavernosometry or cavernosography are rarely done in modern times [15, 25].

## DISCUSSION

The utilization of CPGs is increasing in today's world, and the quality of these guidelines is a major concern for their users [16]. In the current study, which is a scoping review of CPGs related to erectile disorder we have responded to 5 questions related to ED assessment and diagnosis based on the scoping review and 5 select CPGs which had gained higher scores by the research team and the appraisal tool. Overall, the CPGs had desirable overlap in response to the marked questions, nevertheless, there were some differences in details too, which we will discuss below.

Basically, the first step in assessing ED is history-taking, and the CPG published on the UpToDate site in 2020 entitled "Assessment of Male Sexual Disorders" also mentions accurate history-taking as the first step. Important information that should be obtained during sexual history-taking includes assessment of sexual desire, assessment of erectile function, determining the speed of onset of ED and assessment of risk factors and causes. Moreover, it has been stated that the aforementioned information combined with the NPT test can point toward the sexual dysfunction cause [8].

The CPG titled "Erectile Disorders", published by the European Association of Urology (2019) addresses it slightly differently. It states that taking an accurate medical and sexual history of patients – and if present, their spouses – is necessary. It further explains that a sexual history should include information on the patient's sexual orientation, previous and current sexual relations, current emotional status, the onset and duration of the erectile problem, previous consultations and treatments [9].

The guideline published by the American Urological Association (2018) titled "Erectile Disorder" also adds that a socio-psychological history is necessary too, and emphasizes on a complete assessment of the sexual partner and recognizes this as a necessity [15].

The "British Society for Sexual Medicine guidelines on management of erectile dysfunction in men-2017" underscores that it is essential to obtain a detailed history of the problem, including the duration of symptoms and its main related issues. Moreover, attempts should be made to identify the accentuating, predisposing and accelerating factors of the disorder [25]. The Canadian Urological Association's CPG in 2015 also recognizes the diagnostic history as the cornerstone of assessing sexual dysfunction and states that the potential causes of sexual dysfunction include a wide range of organic and medical factors. However, multiple psychological or inter-personal factors (*e.g*., anxiety, depression, distress in relationships) may be among the causative or joint factors [14].

There isn't much discussion on the principles of examining patients with ED, and all select CPGs have addressed the items of complete history-taking, focused physical examination and lab tests. Only the Canadian Urological Association's CPG has mentioned consultations with specialists appropriate to the patient's problem, which seems rational and necessary.

Regarding the utilization of questionnaires to assess ED, based on our scoping review, first, the use of the International Index of Erectile Function (IIEF), and then, the Sexual Health Inventory for Men (SHIM) have been recommended [9, 14]. Routine and primary lab testing should be conducted for these cases, followed by more extensive lab testing appropriate to the patient's complaints and risk factors [8, 9, 14, 25]. There is a difference of opinion on the necessity of assessing individuals' hormonal profiles. The 2020 UpToDate CPG recommends evaluating total testosterone level for assessing sex glands, and the TSH for ruling out thyroid disease in all cases. The American and the European Urological Associations have added the PSA and stated that it may be good for some men affected with ED. If PSA is assessed during ED assessment, suitable consultations should then ensue. Based on the Canadian Urological Association's CPG (2015), the following are considered as complementary lab tests: TSH, LH, FSH (follicle stimulating hormone), prolactin, CBC (complete blood count) and U/A (urine analysis). Assessing these indices is not compulsory in many cases, however, they may be done in accordance with the clinical circumstances.

With respect to the use of special tests for assessing ED in men, most men with ED can be controlled based on their medical and sexual history. On the other hand, some patients may need special diagnostic tests, however, decisions regarding each patient must be made based on his own circumstances [9]. The CPGs examined have recommended special tests for specific individuals, and there are differences of opinion in this respect. The UpToDate site [8] and the American Urological Association [15] have both recommended the NPT in their CPGs to differentiate between the psychogenic and physical causes of ED. However, the Canadian Urological Association's CPG states that the NPT, the sonographic vascular assessment of vessels and other tests are somewhat psychological, and rarely offer beneficial information. The only exceptions are, trauma or other vascular injuries, or the need for legal documentations and/or pharmacological research studies for assessing the effect of a treatment [14].

There are also differences of opinion on the use of invasive tests. For example, the Canadian [14] and European [9] Urological Associations' CPGs state that arteriography and DICC should only be done in patients undergoing vascular reconstructive surgery. However, the CPG published by the American Urological Association in 2018 has emphasized that cavernosometry and cavernography are rarely performed in the modern era. Moreover, these methods are not recommended for obstructive vascular dysfunction [15]. Furthermore, the "British Society for Sexual Medicine guidelines on management of erectile dysfunction in men-2017" states that arteriography should be done only if there is evidence of arterial insufficiency in the doppler sonographic assessment, and cavernosometry is rarely used nowadays [25]. These differences of opinion in utilizing special diagnostic tests underscore the significance of selecting these tests based on the individual's conditions, available equipment, and the goal of diagnosing ED.

## CONCLUSION

This scoping review was conducted on the assessment and diagnosis of erectile disorder in men. Based on our findings, the first and foremost principle in examining the affected individuals is taking a complete detailed history, followed by a physical examination and use of relevant questionnaires to complete the information necessary to diagnose the problem. The next step is to perform routine lab tests; hormonal profile may also be checked, and if need be, special tests will be performed based on an individual's conditions.

Regarding the strengths and limitations of the study, we may refer to the use of a scoping review. Scoping review is a useful method that is increasingly being utilized in evidence synthesis. Researchers may prefer to conduct a scoping review instead of a systematic review when the goal of conducting the review is to examine the current gaps and to clarify the concepts of the study, as it is faster and cheaper and responds to a greater number of questions. The limitation of our study is that the heterogeneity of scoping reviews is very high and a meta-analysis cannot be conducted on them.

## Data Availability

Further data is available from the corresponding author on reasonable request.

## References

[1] Irfan M, Hussain NHN, Noor NM, Mohamed M (2020). Epidemiology of Male Sexual Dysfunction in Asian and European Regions: A Systematic Review. Am J Mens Health.

[2] Gabriel Tobia WWI (2013). DSM-5 Changes in Diagnostic Criteria of Sexual Dysfunctions. Reprod Syst Sex Disord.

[3] Hatzimouratidis K, Amar E, Eardley I, Giuliano F (2010). Guidelines on Male Sexual Dysfunction : Erectile Dysfunction and Premature Ejaculation. Eur Urol.

[4] Rai J, Terry T (2018). Comparison of erectile dysfunction guidelines between the UK (BSSM/NICE) and Europe (EAU). J Clin Urol.

[5] Medina-Polo J, García-Gómez B, Alonso-Isa M, Romero-Otero J (2020). Clinical guidelines on erectile dysfunction surgery: EAU-AUA perspectives. Actas urol Esp.

[6] Peter J, Snyder RCR (2020). Overview of male sexual dysfunction.

[7] KARL T, REW JJH (2016). Erectile dysfunction. Am Fam Physician.

[8] Khera M Evaluation of Male Sexual Dysfunction.

[9] Hatzimouratidis K, Giuliano F, Moncada I, Muneer A (2019). EAU Guidelines on Erectile Dysfunction, Premature Ejaculation, Penile Curvature and Priapism. European Association of Urology Guidelines, Eur Assoc Urol.

[10] Okey-Ewurum I, Amadi A, Nwoke E (2020). Association of Erectile Dysfunction with Systemic Hypertension and Diabetes Mellitus in Rivers State, Nigeria. Int J Res Rev.

[11] Ryu JK, Cho KS, Kim SJ, Oh KJ (2013). Korean Society for Sexual Medicine and Andrology (KSSMA) Guideline on Erectile Dysfunction. World J Mens Health.

[12] Sandoval-Salinas C, Saffon JP, Corredor HA (2020). Quality of Clinical Practice Guidelines for the Diagnosis and Treatment of Erectile Dysfunction: A Systematic Review. J Sex Med.

[13] Ajit Avasthi1, Sandeep Grover TSR (2017). Clinical Practice Guidelines for Management of Sexual Dysfunction. Indian J Psychiatry.

[14] Bella AJ, Lee JC, Carrier S, Bénard F BG (2015). 2015 CUA Practice guidelines for erectile dysfunction. Can Urol Assoc J.

[15] Burnett AL, Nehra A, Breau RH, Culkin DJ (2018). ERECTILE DYSFUNCTION: AUA GUIDELINE. AUA Clin Guidel.

[16] Brouwers MC, Kho ME, Browman GP, Burgers JS (2010). AGREE II: Advancing guideline development, reporting and evaluation in health care. Can Med Assoc J.

[17] Munn Z, Peters MDJ, Stern C, Tufanaru C (2018). Systematic review or scoping review? Guidance for authors when choosing between a systematic or scoping review approach. BMC Med Res Methodol.

[18] Armstrong R, Hall BJ, Doyle J, Waters E (2011). "Scoping the scope" of a cochrane review. J Public Health (Bangkok).

[19] Colquhoun HL, Levac D, O'Brien KK, Straus S (2014). Scoping reviews: Time for clarity in definition, methods, and reporting. J Clin Epidemiol.

[20] Affun-Adegbulu C, Ardalan A (2018). Assessing the problems and developing a scoping review. WHO Guid Res Methods Heal Emerg Disaster Risk Manag.

[21] Eriksen MB, Frandsen TF (2018). The Impact of PICO as a Search Strategy Tool on Literature Search Quality: A Systematic Review. J Med Libr Assoc [Internet].

[22] Cook C (2009). Is clinical gestalt good enough. J Man Manip Ther.

[23] Hoffmann-Eßer W, Siering U, Neugebauer EAM, Brockhaus AC (2017). Guideline appraisal with AGREE II: Systematic review of the current evidence on how users handle the 2 overall assessments. PLoS One.

[24] Brouwers MC, Kho ME, Browman GP, Burgers JS (2010). Development of the AGREE II, part 2: Assessment of validity of items and tools to support application. Can Med Assoc or its Licens.

[25] Hackett G, Kirby M, Wylie K, Heald A (2018). British Society for Sexual Medicine Guidelines on the Management of Erectile Dysfunction in Men-2017. J Sex Med.

